# Prenatal ambient air pollution and maternal depression at 12 months postpartum in the MADRES pregnancy cohort

**DOI:** 10.1186/s12940-021-00807-x

**Published:** 2021-11-27

**Authors:** Theresa M. Bastain, Thomas Chavez, Rima Habre, Ixel Hernandez-Castro, Brendan Grubbs, Claudia M. Toledo-Corral, Shohreh F. Farzan, Nathana Lurvey, Deborah Lerner, Sandrah P. Eckel, Fred Lurmann, Isabel Lagomasino, Carrie Breton

**Affiliations:** 1 Department of Population and Public Health Sciences, USC Keck School of Medicine, University of Southern California, 2001 N. Soto Street, M/C 9237, Los Angeles, CA 90032 USA; 2 Department of Obstetrics and Gynecology, University of Southern California, Los Angeles, USA; 3 Department of Health Sciences, California State University, Northridge, USA; 4 Eisner Health, Los Angeles, USA; 5 Sonoma Technology, Inc, Petaluma, USA; 6 Department of Psychiatry and Behavioral Sciences, University of Southern California, Los Angeles, USA

**Keywords:** MADRES, Maternal health, Depression, Air pollution, Health disparities, Environment

## Abstract

**Background:**

Depression is the leading cause of mental health-related morbidity and affects twice as many women as men. Hispanic/Latina women in the US have unique risk factors for depression and they have lower utilization of mental health care services. Identifying modifiable risk factors for maternal depression, such as ambient air pollution, is an urgent public health priority. We aimed to determine whether prenatal exposure to ambient air pollutants was associated with maternal depression at 12 months after childbirth.

**Methods:**

One hundred eighty predominantly low-income Hispanic/Latina women participating in the ongoing MADRES cohort study in Los Angeles, CA were followed from early pregnancy through 12 months postpartum through a series of phone questionnaires and in-person study visits. Daily prenatal ambient pollutant estimates of nitrogen dioxide (NO_2_), ozone (O_3_), and particulate matter (PM_10_ and PM_2.5_) were assigned to participant residences using inverse-distance squared spatial interpolation from ambient monitoring data. Exposures were averaged for each trimester and across pregnancy. The primary outcome measure was maternal depression at 12 months postpartum, as reported on the 20-item Center for Epidemiologic Studies-Depression (CES-D) scale. We classified each participant as depressed (*n* = 29) or not depressed (*n* = 151) based on the suggested cutoff of 16 or above (possible scores range from 0 to 60) and fitted logistic regression models, adjusting for potential confounders.

**Results:**

We found over a two-fold increased odds of depression at 12 months postpartum associated with second trimester NO_2_ exposure (OR = 2.63, 95% CI: 1.41–4.89) and pregnancy average NO_2_ (OR = 2.04, 95% CI: 1.13–3.69). Higher second trimester PM_2.5_ exposure also was associated with increased depression at 12 months postpartum (OR = 1.56, 95% CI: 1.01–2.42). The effect for second trimester PM_10_ was similar and was borderline significant (OR = 1.58, 95% CI: 0.97–2.56).

**Conclusions:**

In a low-income cohort consisting of primarily Hispanic/Latina women in urban Los Angeles, we found that prenatal ambient air pollution, especially mid-pregnancy NO_2_ and PM_2.5_, increased the risk of depression at 12 months after childbirth. These results underscore the need to better understand the contribution of modifiable environmental risk factors during potentially critical exposure periods.

**Supplementary Information:**

The online version contains supplementary material available at 10.1186/s12940-021-00807-x.

## Background

Depression is the leading cause of mental health-related morbidity worldwide, affecting approximately 300 million people annually [1]. While effective therapies are available, depression often requires chronic treatment with significant healthcare costs [2, 3]. Depression is nearly twice as prevalent among women than men [4, 5]. This difference is apparent early in adolescence and continues through midlife and beyond [6], suggesting that the reproductive years are particularly important for risk of depression in women.

Prevalence rates of depression are similar for Hispanic, non-Hispanic white and Black communities in the US [7]; however, reports of depressive symptoms have been higher in Hispanic populations than other groups [8]. The American Psychiatric Association has acknowledged that Hispanic communities have substantial barriers to accessing mental health services including limited knowledge about symptoms of mental health problems, cultural stigma associated with mental illness, lack of health insurance or inadequate coverage, and limited availability of culturally-competent and bilingual mental health care professionals [9].

Exposure to outdoor air pollution disproportionately impacts socio-economically disadvantaged populations and Hispanic and Black communities [10–13]. Increasing evidence suggests that pregnancy is a vulnerable window of exposure for later maternal health effects [14] including depression and other mental health disorders. Two recent studies showed that gestational exposure to particulate matter less than 2.5 μm in aerodynamic diameter [PM_2.5_] was associated with increased postpartum depressive symptoms [15, 16]. In particular, exposure during mid-pregnancy—a period characterized by rapid rises in cardiac output, maternal blood volume, heart rate and pulmonary circulation necessary to maintain sufficient blood supply to the developing fetus [17]—was shown to be associated with increased postpartum anhedonia symptoms at 6 or 12 months after childbirth particularly among Black women [15]. While there are multiple established biological and psychosocial risk factors for depression [18, 19], identifying potentially modifiable environmental risk factors to protect vulnerable populations is an urgent public health priority.

In this study, we examined whether higher concentrations of ambient air pollutants (including nitrogen dioxide [NO_2_], ozone [O_3_], PM_2.5_ and PM_10,_), during pregnancy were associated with increased depression at 12 months after childbirth in the MADRES cohort.

## Methods

### Study design overview

The MADRES cohort is an ongoing prospective pregnancy cohort that recruited over 800 predominantly low-income Hispanic/Latina women at community health prenatal care providers in Los Angeles, CA between 2015 and 2020. The protocol, study design and detailed study procedures for the MADRES cohort are described elsewhere [20]. Briefly, prenatal data were collected during each trimester by interviewer-administered questionnaires in English or Spanish on the phone or in person. Follow-up of mothers and infants through 12 months postpartum consisted of a series of five phone questionnaires and in-person visits.

The full study protocol was approved by the University of Southern California Institutional Review Board and informed consent and HIPAA authorization for medical records abstraction were obtained prior to participation by bilingual MADRES staff members.

### Participants

**Fig. 1:**
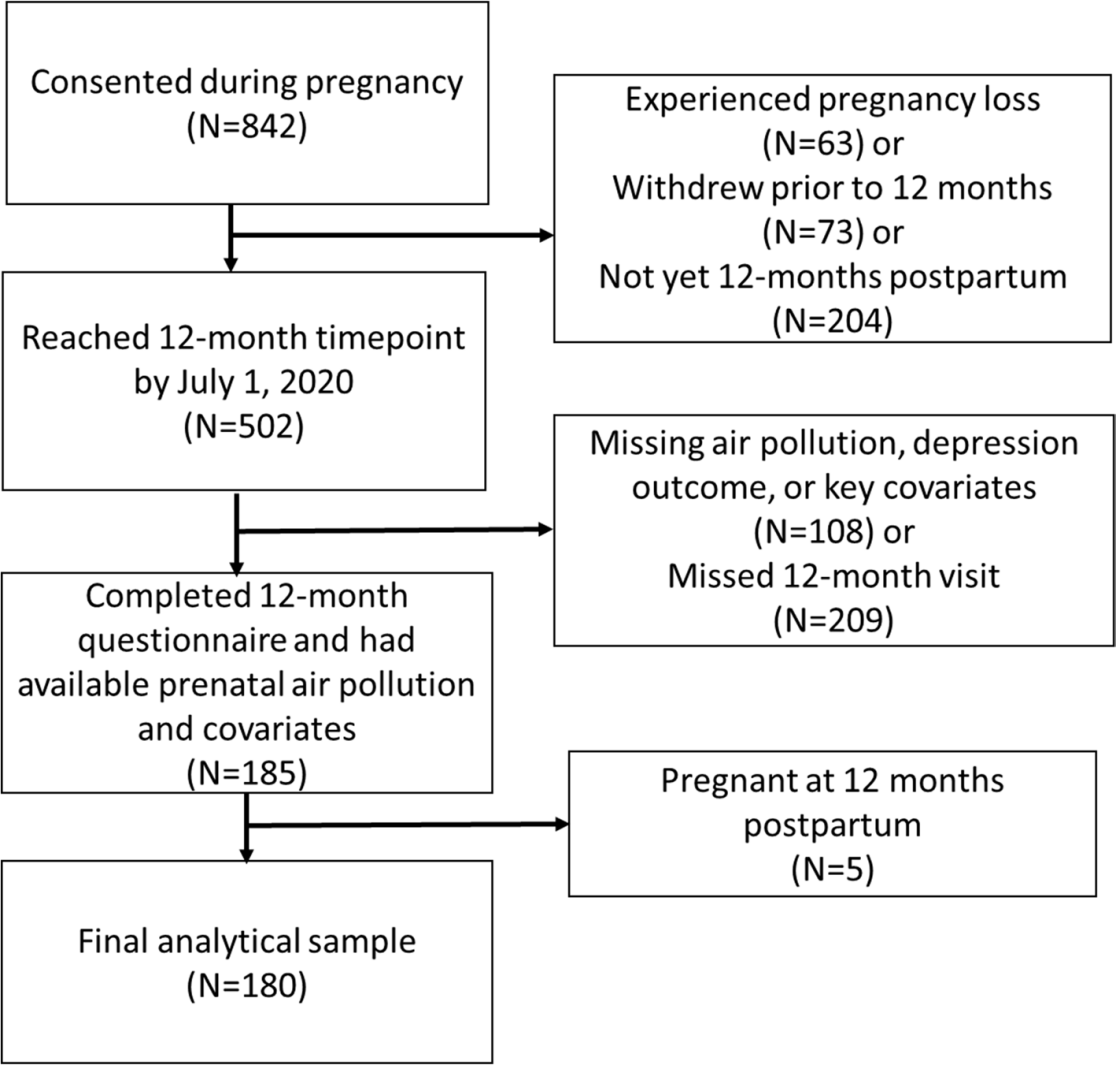
Consort Diagram Illustrating Data Availability

Study inclusion criteria for the MADRES cohort included < 30 weeks gestation, singleton pregnancy, ≥18 years old, and English or Spanish speaking. Study exclusions included current incarceration, HIV positive status and any cognitive or physical disability that would prevent participation in study procedures or providing informed consent. The consort diagram illustrating data available for this analysis is shown in Fig. 1. Participants in this sample were similar to the overall cohort (see Supplemental Table 1). The final sample included 180 participants who had completed the questionnaire at 12 months, were not pregnant, had key covariate information during pregnancy and had available prenatal ambient air pollution assignments for at least one pollutant.

### Depression outcome assessment

#### Center for epidemiological studies-depression (CES-D) scale

The CES-D is a widely-used, validated 20-item instrument that was developed for use in epidemiological studies for assessing depressive symptoms experienced within the past week [21]. Prior studies have shown stability of CES-D scores in the first year postpartum and linkages with diagnosis of depression [22, 23]. Items cover multiple symptom clusters including depressed mood, loss of interest, feelings of guilt and worthlessness, psychomotor dysfunction, loss of appetite, and sleep disturbance. Scores range from 0 to 60 and scores 16 or above indicate risk for clinical depression [24]. The CES-D is a reliable measure for evaluating depressive symptoms among women and men, Hispanic and non-Hispanic populations, and across age groups [21, 25, 26]. We classified a participant as depressed using the recommended cutoff of 16 or above, which is suggestive of clinical depression [21].

Previous history of depression was defined as depression diagnosis or history of antidepressant medication documented on the prenatal medical record.

### Ambient air pollution exposure assessment

#### Residential address timelines

Residential address history and occupancy dates from 1 year prior to conception through the third trimester of pregnancy were completed at home by study participants prior to the third trimester visit and then subsequently discussed with study staff to ensure data were captured accurately. Prospective address collection occurred at each follow-up study timepoint to capture residential mobility. Addresses were geocoded and daily residential timelines were assembled for each participant.

#### Ambient air pollution

Daily estimates of NO_2_, O_3,_ PM_2.5_ and PM_10_ were assigned to the locations for the gestational time periods relevant for each study participant, using inverse-distance-squared weighted spatial interpolation from ambient air quality monitoring data (U.S. EPA Air Quality System). Southern California has one of the densest monitoring networks in the US. The average number of monitors used in estimating concentrations at each residential location in our study was 3.97 for NO_2_ and O_3_ (range 2.04–4.00), 3.98 for PM_2.5_ (range (2.20–4.00) and 3.83 for PM_10_ (range 1.37–4.00). The spatial distribution of NO_2_ monitors in the monitoring network in Los Angeles overlayed on MADRES participant addresses at study enrollment is depicted in Supplement Fig. 1. We calculated average pollutant concentrations for each participant by trimester and across pregnancy.

#### Daily temperature

Daily temperature was calculated as the average of daily maximum and minimum temperatures extracted from a high-resolution (4 km × 4 km) gridded surface meteorological dataset developed by Abatzoglou [27].

### Covariates

A wide range of data were collected via interviewer-administered questionnaires, including preconception personal and family health history, pregnancy history, proxy measures of acculturation and demographic information. Questionnaire data on possible indoor air pollution sources, mode of travel and commuting time; housing characteristics; and history of tobacco smoke exposure were also collected. Prenatal medical records and birth records were abstracted at participating clinic sites and delivery hospitals for medication use during pregnancy, prenatal health history, delivery outcomes, and birth complications.

### Statistical methods

#### Descriptive and univariate analyses

We summarized the distributions of each pollutant by trimester and over the entire pregnancy and calculated Spearman correlation coefficients to examine their correlations. We fitted unadjusted logistic regression models to explore relationships between depression and key demographic characteristics and maternal health conditions. We also examined differences between “depressed” and “not depressed” participants at 12 months using: independent t-tests for normally-distributed continuous variables, Wilcoxon Mann-Whitney tests for non-normally distributed variables, and Pearson’s Chi-Square tests for categorical variables (or Fisher’s Exact test if cell counts < 5).

#### Multivariable logistic regression

We fitted multivariable logistic regression models to evaluate associations between trimester-specific and pregnancy average exposures and depression at 12 months postpartum. We considered a wide range of potential confounders identified a priori using Directed Acyclic Graphs (DAGs) that could be associated with both exposure and outcome including history of depression or antidepressant medication use, proxy measures of acculturation (language preference, nativity, years living in the US), demographic information (age, race, ethnicity, household income, education) and housing characteristics (air conditioning use, opening of windows, building or residence type and age). We also evaluated effects of indoor air pollution sources (cooking frequency and duration, gas stove usage); mode of travel and commuting time to reduce exposure measurement error; history of tobacco smoke exposure (lifetime, prenatal and second-hand); and personal and family health history, pregnancy history (e.g., parity, gestational age at birth, delivery type, pregnancy or birth complications).

We evaluated each of these potential covariates for confounding and included them in our final models if they were known or hypothesized to be associated with both exposure and outcome (age, ethnicity, nativity, household income, education, previous history of depression diagnosis, air conditioning use), if they resulted in greater than a 10% change in the exposure effect estimates (average temperature), or if they were a study design variable that could account for unmeasured residual confounding by socioeconomic factors (study recruitment site). We parameterized the covariates included in the models as follows: maternal age at consent (continuous, in years); average temperature over the exposure averaging period (continuous, in degrees Celsius); recruitment site (indicators for recruitment site locations); maternal ethnicity and nativity (non-Hispanic; US born Hispanic; foreign born Hispanic-Mexico; foreign born Hispanic-other); maternal income (less than $15,000; $15,000–$29,999; $30,000–$49,999; $50,000–$99,999; $100,000+; don’t know); air conditioning use during pregnancy (yes/no); depression diagnosis on medical record (yes/not recorded); and maternal education level (less than 12th grade; completed grade 12; some college or technical school; completed 4 years of college; some graduate training after college).

Participants with available data for any of the ambient pollutants and depression outcome at 12 months were included. Missing values for any of the categorical covariates were included in the multivariable models with missing indicators. We accounted for small sample bias in maximum likelihood estimation, by fitting logistic regression models with and without Firth’s correction method for penalized likelihood [28, 29]. Exposure effect estimates were scaled to one standard deviation of exposure to each trimester-specific or pregnancy-average pollutant. We also conducted sensitivity analyses to determine whether (1) previous history of depression influenced our estimates; (2) our final model estimates were sensitive to residual confounding by population-level (census tract) socioeconomic and vulnerability factors not accounted for by individual level covariates; and (3) our results were influenced by annual average postpartum (birth to 12 months postpartum) air pollution levels. All analyses assumed a two-sided alternative hypothesis, an alpha level of 0.05 and were conducted using SAS Version 9.4.

## Results

Demographic, maternal health characteristics and delivery outcomes are presented in Table 1. Participants in this sample were on average 29.8 years of age at study entry (SD = 6.0 years). The majority of participants self-identified as Hispanic (79%) and 39% of participants were born outside of the United States. Most participants reported being married or living together with a partner (73%). Fifty percent reported annual household income less than $30,000 and 52% of participants had at most a 12th grade education. Twenty-one women (12%) had a previous history of depression.

**Table 1:** Demographic characteristics, maternal health conditions and predictors of maternal depression at 12 months postpartum

Characteristic	N	Mean (SD), %	OR	95% CI	
**Age at Study Entry (Years)**	180	29.8 (6.0)	1.00	0.94	1.07
**Race and Ethnicity**					
Hispanic	143	79.4%	-- REF --		
Black	8	4.4%	1.83	0.35	9.68
Non-Hispanic White	21	11.7%	0.92	0.25	3.38
Multiracial	2	1.1%	5.50	0.33	91.26
Other	5	2.8%	1.38	0.15	12.89
Missing	1	0.6%			
**Ethnicity and Nativity**					
US-Born Hispanic	64	37.40%	-- REF --		
Foreign-Born Hispanic	71	41.50%	1.83	0.68	4.91
Non-Hispanic	36	21.10%	1.97	0.63	6.14
Missing	9	5.00%			
**Annual Household Income at Study Entry**					
Less than $15,000	40	22.2%	-- REF --		
$15,000 to $29,999	51	28.3%	1.01	0.34	3.00
$30,000 to $49,999	22	12.2%	0.74	0.17	3.22
$50,000 to $99,999	12	6.7%	0.43	0.05	3.88
$100,000 or more	16	8.9%	1.09	0.24	4.86
Don’t Know	38	21.1%	0.88	0.27	2.92
Missing	1	0.6%			
**Educational Attainment at Study Entry**					
< 12th Grade	52	28.9%	-- REF --		
Completed 12th Grade	42	23.3%	1.53	0.47	4.97
Some College or Technical School	43	23.9%	2.32	0.77	7.03
Completed College	24	13.3%	0.33	0.04	2.94
Some Graduate Training	18	10.0%	2.95	0.77	11.23
Missing	1	0.6%			
**Marital Status**					
Never married, single	31	17.2%	-- REF --		
Married	60	33.3%	1.19	0.34	4.23
Living together	71	39.4%	1.51	0.45	5.07
Divorced or separated	5	2.8%	1.69	0.15	19.16
Missing	13	7.2%			
**Pre-Pregnancy BMI Category**					
Normal	46	25.6%	-- REF --		
Underweight	2	1.1%	3.60	0.21	62.81
Overweight	67	37.2%	0.42	0.15	1.20
Obese	65	36.1%	0.73	0.28	1.91
**Parity**					
1st born	65	36.1%	-- REF --		
2nd born	48	26.7%	0.98	0.36	2.66
3rd born or more	58	32.2%	0.79	0.29	2.11
Missing	9	5.0%			
**Mode of Delivery**					
Vaginal	132	73.3%	-- REF --		
Planned C-Section	22	12.2%	1.00	0.31	3.22
Unplanned/Emergency C-Section	26	14.4%	0.18	0.02	1.39
**Gestational Age at Delivery**					
Preterm (< 37 weeks)	22	12.2%	0.43	0.09	2.02
Early Term (37 weeks–38 weeks 6/7 days)	47	26.1%	0.63	0.23	1.72
Full Term (39 weeks–40 weeks 6/7 days)	96	53.3%	-- REF --		
Late Term or Post Term (≥41 weeks	15	8.3%	1.08	0.28	4.24
**IOM Weight Gain Recommendations**					
Adequate	54	30.0%	-- REF --		
Excessive	74	41.1%	1.43	0.53	3.87
Insufficient	34	18.9%	0.90	0.24	3.32
Missing	18	10.0%			
**Hypertensive Disorders**					
Normotensive	147	81.7%	-- REF --		
Hypertensive	32	17.8%	0.70	0.23	2.16
Missing	1	0.6%			
**Diabetic Disorders**					
Normal	118	65.6%	-- REF --		
Glucose Intolerant	35	19.4%	0.69	0.24	1.97
Gestational Diabetes (GDM)^a^	18	10.0%	–	–	–
Chronic Diabetes	8	4.4%	0.59	0.07	5.04
Missing	1	0.6%			
**Depression Diagnosis or Antidepressant Medication**					
No	159	88.3%	-- REF --		
Yes	21	11.7%	**3.11**	**1.13**	**8.57**

^a^All participants with GDM scored < 16 on CES-D

There were a few demographic differences between this analytic sample of 180 women and the full cohort of 842 recruited women (Supplemental Table 1). The participants in this sample were slightly older at study entry compared to the full cohort (mean age 29.8 years versus 28.4 years). There were modest differences in socioeconomic status with respect to maternal educational attainment and household income level, with the current analytic sample having a higher proportion of college graduates or above (23.3% versus 14.1% overall) and a higher proportion of participants reporting household income greater than $50,000 per year (15.6% versus 9.2% overall) reported household income on average. However, there are a greater percentage of missing data in the overall cohort relative to the analytic sample for these characteristics. In addition, 11.7% of the analytical sample had a documented diagnosis of depression or antidepressant medication use documented on their medical record compared to 7.0% in the overall cohort.

Twenty-nine participants reported symptoms on the CES-D scale consistent with depression (score of ≥16). The distribution of scores on the CES-D is shown in Supplemental Fig. 2. We examined the relationship of depression at 12 months postpartum with multiple demographic characteristics and maternal health conditions and found the only significant predictor of maternal depression at 12 months was a previous history of depression (OR = 3.11, 95% CI: 1.13–8.57) (Table 1). We saw suggestive differences by nativity among Hispanic participants. Relative to US-Born Hispanic participants, foreign-born Hispanic participants had higher odds of depression (OR = 1.83, 95% CI 0.68–4.97).

Average daily ambient pollutant (NO_2_, O_3,_ PM_2.5_, and PM_10_) concentrations over each trimester and across pregnancy are shown in Table 2. Across pregnancy, average daily NO_2_ concentrations were 17.0 ppb (SD = 2.7 ppb), O_3_ concentrations were 26.1 ppb (SD = 2.7 ppb), PM_2.5_ concentrations were 12.2 μg/m^3^ (SD = 1.1 μg/m^3^), and PM_10_ concentrations were 30.9 μg/m^3^ (SD = 3.8 μg/m^3^).

**Table 2:** Ambient pollutant concentrations over four prenatal averaging periods

Pollutant	1st trimester		2nd trimester		3rd trimester		Pregnancy average	
	Mean	SD	Mean	SD	Mean	SD	Mean	SD
**NO**_**2**_**(ppb)**	16.2	5.9	17.4	5.2	17.4	5.7	17.0	2.7
**O**_**3**_**(ppb)**	27.3	6.2	25.7	5.9	25.4	6.8	26.1	2.7
**PM**_**2.5**_**(μg/m**^**3**^**)**	12.3	2.0	12.4	2.0	12.1	2.7	12.2	1.1
**PM**_**10**_**(μg/m**^**3**^**)**	31.2	5.1	31.0	5.9	30.0	6.1	30.8	3.7

Ozone (O_3_) and NO_2_ showed significant negative correlations across each of the exposure averaging periods (e.g., 2nd trimester *r* = − 0.86, *p* < 0.0001). PM_2.5_, PM_10_ and NO_2_ were positively correlated across each of the exposure averaging periods, although the correlations were strongest between PM_10_ and PM_2.5_ concentrations (e.g., 2nd trimester *r* = 0.87, *p* < 0.0001). Within pollutant, correlations across trimesters varied with generally positive correlations seen between 1st and 2nd trimesters and negative correlations between 1st and 3rd trimesters (see Supplemental Fig. 3).

Table 3 shows unadjusted and adjusted odds ratios for each ambient pollutant for trimester-specific and average pregnancy models. Table 3 also shows each adjusted model with Firth’s method applied to account for small sample bias for maximum likelihood estimation.

**Table 3:** Association of prenatal ambient air pollutants with maternal depression at 12 months postpartum

Pollutant	Exposure Averaging Period	Model 1: Unadjusted		Model 2: Adjusted^**b**^		Model 3: Adjusted^**b**^ with Firth’s Correction	
		N	OR^**a**^ (95% CI)	N	OR (95% CI)	N	OR (95% CI)
**NO**_**2**_**(ppb)**	Trimester 1	179	1.02 (0.68, 1.53)	179	1.29 (0.70, 2.40)	179	1.24 (0.72, 2.14)
	Trimester 2	**180**	1.82 (1.22, 2.73)**	**180**	2.63 (1.41, 4.89)**	**180**	2.22 (1.28, 3.85)**
	Trimester 3	179	1.03 (0.69, 1.53)	179	0.91 (0.55, 1.49)	179	0.92 (0.59, 1.44)
	Across Pregnancy	**180**	1.58 (1.05, 2.37)*	**180**	2.04 (1.13, 3.69)*	**180**	1.82 (1.08, 3.07)*
**O**_**3**_**(ppb)**	Trimester 1	179	0.89 (0.60, 1.33)	179	0.60 (0.31, 1.16)	179	0.65 (0.37, 1.16)
	Trimester 2	180	0.75 (0.51, 1.12)	180	0.68 (0.35, 1.32)	180	0.73 (0.40, 1.31)
	Trimester 3	179	1.14 (0.76, 1.70)	179	1.48 (0.84, 2.63)	179	1.40 (0.84, 2.35)
	Across Pregnancy	180	0.87 (0.57, 1.32)	180	0.88 (0.53, 1.47)	180	0.90 (0.57, 1.45)
**PM**_**2.5**_**(μg/m**^**3**^**)**	Trimester 1	180	0.79 (0.52, 1.20)	180	0.80 (0.47, 1.35)	180	0.86 (0.54, 1.36)
	Trimester 2	180	1.40 (0.95, 2.06)	**180**	1.56 (1.01, 2.42)*	180	1.47 (0.98, 2.19)
	Trimester 3	179	0.94 (0.63, 1.41)	179	0.93 (0.57, 1.51)	179	0.95 (0.61, 1.47)
	Across Pregnancy	180	1.11 (0.75, 1.64)	180	1.33 (0.83, 2.15)	180	1.28 (0.82, 1.99)
**PM**_**10**_**(μg/m**^**3**^**)**	Trimester 1	179	0.88 (0.59, 1.31)	179	0.69 (0.37, 1.28)	179	0.76 (0.44, 1.30)
	Trimester 2	180	1.34 (0.92, 1.97)	180	1.58 (0.97, 2.56)	180	1.46 (0.94, 2.28)
	Trimester 3	179	1.03 (0.69, 1.53)	179	1.02 (0.58, 1.80)	179	1.02 (0.61, 1.70)
	Across Pregnancy	180	1.17 (0.79, 1.73)	180	1.27 (0.80, 2.04)	180	1.22 (0.79, 1.88)

*p < 0.05; **p < 0.01

^a^All odds ratios (OR) are scaled to one SD in exposure over the averaging period for each pollutant

^b^Adjusted for recruitment site, maternal age, ethnicity by nativity, household income, education, air conditioning use during pregnancy, previous history of depression, and average temperature over the exposure averaging period

We found that higher second trimester ambient NO_2_ was associated with increased depression at 12 months in unadjusted models (OR = 1.82, 95% CI: 1.22–2.73). In adjusted models, we found a 2.6 fold increased odds of depression at 12 months postpartum associated with second trimester NO_2_ exposure (OR = 2.63, 95% CI: 1.41–4.89). When we applied Firth’s method, the effect estimate was mildly attenuated (OR = 2.22, 95% CI: 1.28–3.85), but remained statistically significant. We also found significantly increased odds of depression associated with pregnancy-average NO_2_ (OR = 2.04, 95% CI: 1.13–3.69) in adjusted models. Neither first nor third trimester NO_2_ concentrations were associated with depression at 12 months.

We found no significant associations between prenatal ambient ozone concentrations and depression at 12 months postpartum. For example, second trimester O_3_ was inversely associated with depression at 12 months, although this relationship was not significant (OR = 0.68, 95% CI: 0.35–1.32).

Similar to our findings for second trimester NO_2_ exposure, we found that higher second trimester PM_2.5_ exposure was associated with increased depression at 12 months postpartum in the fully adjusted model (OR = 1.56, 95% CI: 1.01–2.42). The effect estimate was slightly attenuated with Firth’s correction (OR = 1.47, 95% CI: 0.98–2.10) and the association remained marginally significant. Pregnancy average PM_2.5_ exposure showed increased depression at 12 months (OR = 1.33, 95% CI: 0.83–2.15), although this association did not reach statistical significance. Neither first nor third trimester PM_2.5_ concentrations were significantly associated with depression at 12 months.

While not statistically significant, the pattern of effects was similar for prenatal PM_10_. Both second trimester PM_10_ exposure (OR = 1.58, 95% CI: 0.97–2.56) and pregnancy average exposure (OR = 1.27, 95% CI: 0.80–2.04) were associated with increased odds of depression in adjusted models.

We examined whether a previous history of depression influenced our results. When we removed the 21 participants with a previous diagnosis of depression, results were similar (e.g., OR_2nd trimester NO2_ = 2.35, 95% CI: 1.21–4.55; data not shown). We also conducted a sensitivity analysis adjusting for CalEnviroScreen 3.0 [30] Population Characteristics scores (census tract-level population socioeconomic vulnerability) assigned to each participant’s study enrollment address to investigate whether there could be residual confounding by neighborhood level socioeconomic factors not accounted for by adjustments for household income, maternal education and study recruitment site. We found our results to be robust to the addition of population vulnerability scores (e.g., OR_2nd trimester NO2_ = 2.63, 95% CI:1.40, 4.93; Supplemental Table 2). Similarly, when we adjusted for 12 month average postpartum pollutant levels, our results were largely unchanged (e.g., OR_2nd trimester NO2_ = 2.70, 95% CI:1.43, 5.12; Supplemental Table 3).

## Discussion

In a cohort of primarily low-income Hispanic/Latina women in Los Angeles, we found that exposure to prenatal ambient air pollution increased the risk of depression at 1 year after childbirth. Specifically, we found that second trimester and pregnancy average ambient NO_2_ levels were associated with greater than a two-fold increased risk of maternal depression at 12 months postpartum. We also found that second trimester PM_2.5_ concentrations were associated with increased depression at 12 months and the pattern of effects was similar for second trimester ambient PM_10_ concentrations.

We investigated whether the second trimester could be a proxy for the more proximate exposure period to the postpartum outcome measurement (e.g., exposure at 6–9 months postpartum). While monthly pollutant levels tended to be correlated year to year, we found no evidence that NO_2_ exposure 6–9 months postpartum was associated with increased depression at 12 months (data not shown). Mounting evidence suggests that pregnancy is a vulnerable window of exposure for later maternal health effects [14]. Specifically, the second trimester could be a vulnerable period for both behavioral and physiological reasons. The second trimester is often characterized by decreased nausea, better sleep patterns and an increased energy level that may encourage greater time spent outdoors and result in higher ambient air pollution exposures. Moreover, beginning in the second trimester, the uterus expands and rapid rises in cardiac output, maternal blood volume, heart rate and pulmonary circulation occur that are necessary to maintain sufficient blood supply to the developing fetus [17].

Multiple human and animal studies have shown that air pollutants affect the brain through neuroinflammatory pathways [31–33]. The hypothalamic-pituitary-adrenal (HPA) axis can be activated by inhaled air pollution [34]. Prenatal PM_10_ and PM_2.5_ exposures have been linked to elevated maternal CRP and Il-6 levels [35, 36] and considerable evidence implicates these neuroinflammatory pathways in depression pathophysiology [37–42]. Pregnancy could be a specific vulnerable period for air pollution exposure effects on neuroinflammatory pathways as normal adaptive changes to the respiratory system, such as incremental gestational increases in respiration rate, minute ventilation and oxygen consumption, accommodate the metabolic demands of pregnancy and oxygen transfer across the placenta [17]. In addition, pregnant women have greater blood concentrations of inhaled compounds [43, 44], suggesting that pregnancy maybe a particularly vulnerable period for neurological effects of inhaled ambient air pollutants.

Our results are consistent with two recent studies that showed significant effects of PM_2.5_ exposure on maternal postpartum depression [15, 16]. The first study in Boston, MA showed that second trimester PM_2.5_ exposure was associated with increased postpartum anhedonia symptoms at 6 or 12 months after childbirth and the effects were most pronounced among Black women [15]. They did not find significant effects within Hispanic women alone; however, the population of Hispanic women in the Boston study was largely born in the United States, with few women born in Mexico or of Mexican origin [16]. In contrast, nearly 50% of Hispanic women in the MADRES cohort were born outside of the U.S.—the majority of whom were born in Mexico (data not shown). The second study conducted in Mexico City found that a 5-μg/m^3^ increase in average prenatal PM_2.5_ exposure was associated with an 83% increase in risk of postpartum depression at 6 months. Among women without postpartum depression at 1 month, the same exposure showed a 158% increase in risk of late-onset postpartum depression at 6 months, suggesting that the detrimental impacts of prenatal PM_2.5_ exposure may not occur until several months following childbirth [16].

There is an increasing body of evidence documenting the impact of chronic air pollution exposure on major depression [45, 46]. In a recent study of more than 150 million individuals in the US and Denmark, air pollution was associated with higher rates of major depression in both countries [47]. Long-term exposure to air pollution was also associated with increased depression in a study of middle-age and older adults in Spain [48] and in with new-onset depression among midlife and older women in the US [49].

It is increasingly acknowledged that exposure to air pollution disproportionately impacts health disparity populations [10–12]. In California, Black and Hispanic communities bear the highest burden of exposure to multiple environmental chemicals [30, 50]. Approximately 80% of MADRES participants are Hispanic and about 50% were born outside of the US. Hispanic immigrant women have several unique risk factors for depression, including separation from family members and feelings of isolation [51]. While prevalence rates of depression are similar for U.S. Hispanic, non-Hispanic white and Black communities [7], reporting of depressive symptoms has been higher among Hispanic populations [8]. One reason suggested for the lower reported prevalence rate relative to reported symptoms is that the Hispanic community underutilizes mental health care services [52–54].

Our results suggested that foreign-born Hispanic participants had a higher risk of depression at 12 months than US-Born Hispanic participants and this increased risk varied by country of birth (data not shown). Multiple studies have shown that mental health status among immigrant populations declines with increasing acculturation [55–57]. Studies have also suggested that postpartum depression among US Hispanic immigrant populations is 40–60% compared to ~ 14% of all new mothers in the US [58–60]. Premigration stress, low socioeconomic status, lack of social support, stressful life events, language barriers and difficulty adapting to a new culture may contribute to this disparity in postpartum depression [61].

This is the first study to our knowledge that has investigated the effects of multiple prenatal ambient pollutants on later maternal depression in a diverse population using daily estimates of ambient air pollutant exposures tied to time-resolved residential timelines, accounting for residential mobility. Our findings were robust to multiple sensitivity adjustments including population-level social vulnerabilities and postpartum air pollution levels. We used a validated depression scale with demonstrated reliability among women and men, Hispanic and non-Hispanic populations, and across age groups [21, 25, 26]. In addition, we were able to evaluate the impact of prior depression diagnosis and antidepressant medication usage as well as leverage considerable symptom data obtained from questionnaires. We found that removing women with a prior depression diagnosis did not impact our findings even though women with depression may be encouraged to increase their physical activity during pregnancy to reduce depressive symptoms [62–65] and increased physical activity could lead to increased exposure to ambient air pollution [66].

As with all studies, there were some limitations to our approach. Our findings may not be generalizable to all populations as the MADRES cohort draws its participants from prenatal health clinics in areas of high urban air pollution that serve women who have traditionally had lower access to health care—especially, mental health care—services. It has also been suggested that our measure of depression—the CES-D scale—may overestimate the number of true cases of depression [26]. However, we have no reason to assume the outcome misclassification would be related to air pollution exposure, suggesting that any bias would be toward the null. We also did not have information on whether our participants were diagnosed with postpartum depression, or whether those with a history of depression were undergoing treatment at 12 months. In fact, among the 21 women with a documented history of depression, only 7 women reported symptoms consistent with depression at 12 months, suggesting that the remaining women with a history of depression may have controlled their symptoms effectively with antidepressant medication or psychotherapy.

While retention in the MADRES cohort has remained high overall, participants are not always able to complete all study timepoints and therefore the available outcome follow-up data at 12 months was limited to a relatively small sample. We found modest differences in sociodemographic characteristics between the participants in this subsample and the overall cohort and therefore cannot indefinitely rule out whether characteristics related to participation could have influenced our results. We also investigated whether the women who missed the 12-month postpartum study visit had different patterns of prenatal exposure to ambient air pollutants from those who completed the visit. We found that the average prenatal pollutant concentrations were not significantly different for women who completed and did not complete this visit (data not shown) and this was unlikely to account for our findings.

While we did not have measurements of personal exposure to air pollution, we estimated exposure to ambient air pollution at each participant’s home based on inverse distance weighting methods. These methods are likely to result in exposure measurement error, in part due to the fundamental assumption that participants’ exposures occur primarily outdoors at the home. However, this error applies equally to depression cases and non-cases, suggesting that any bias would also be toward the null. Even with these limitations, our results were robust to multiple adjustments, and multiple correlated ambient pollutants exhibited similar effects on maternal depression at 12 months. Further investigations are needed to examine the underlying biological mechanisms at play and to explore specific mid-pregnancy windows of susceptibility to effects of air pollution on women’s postpartum health.

## Conclusions

We found compelling evidence that prenatal ambient air pollution exposure may have long-term impacts on maternal depression. These results underscore the need to better understand the contribution of modifiable environmental risk factors during susceptible periods, and have important implications for identification, prevention and treatment of depression in vulnerable women in the years following childbirth.

## Supplementary Information

**Additional file 1: Supplement Figure 1.** Monitoring network density for nitrogen dioxide (NO_2_) in Los Angeles, CA.

**Additional file 2: Supplement Figure 2.** Distribution of Scores on the Center for Epidemologic Studies-Depression (CES-D) Scale at 12-Months Postpartum.

**Additional file 3: Supplement Figure 3.** Spearman Correlations for each Ambient Pollutant by Trimester and Across Pregnancy.

**Additional file 4: Supplement Table 1.** Demographic characteristics in the full MADRES cohort and the current analytic sample. **Supplement Table 2.** Association of Prenatal Ambient Air Pollutants with Maternal Depression at 12 Months Postpartum, Additionally Adjusted for CalEnviroScreen Population Vulnerability Score. **Supplement Table 3.** Association of Prenatal Ambient Air Pollutants with Maternal Depression at 12 Months Postpartum, Additionally Adjusted for 12 Month Annual Average Postpartum Ambient Air Pollution.

## Data Availability

The datasets used and/or analyzed during the current study are available from the corresponding author on reasonable request and after approval by the USC Institutional Review Board.

## Abbreviations

MADRES: Maternal And Developmental Risks from Environmental and Social Stressors cohort study
NHANES: National Health and Nutrition Examination Survey
HIPAA: Health Information Portability and Accountability Act
USC: University of Southern California
CES-D: Center for Epidemiological Studies-Depression Scale
PM_2.5_: Particulate Matter less than 2.5 μm in diameter
PM_10_: Particulate Matter less than 10 μm in diameter
NO_2_: Nitrogen dioxide
O_3_: Ozone
HPA: Hypothalamic-pituitary-adrenal axis
GA: Gestational age
BMI: Body mass index
