# Supplementary figures and images for: Prenatal ambient air pollution and maternal depression at 12 months postpartum in the MADRES pregnancy cohort
Source: Environ Health. 2021 Nov 27;20:121. doi: 10.1186/s12940-021-00807-x (PMC8626870; doi:10.1186/s12940-021-00807-x)

**Supplement Figure 1. Monitoring network density for nitrogen dioxide (NO2) in Los Angeles, CA**


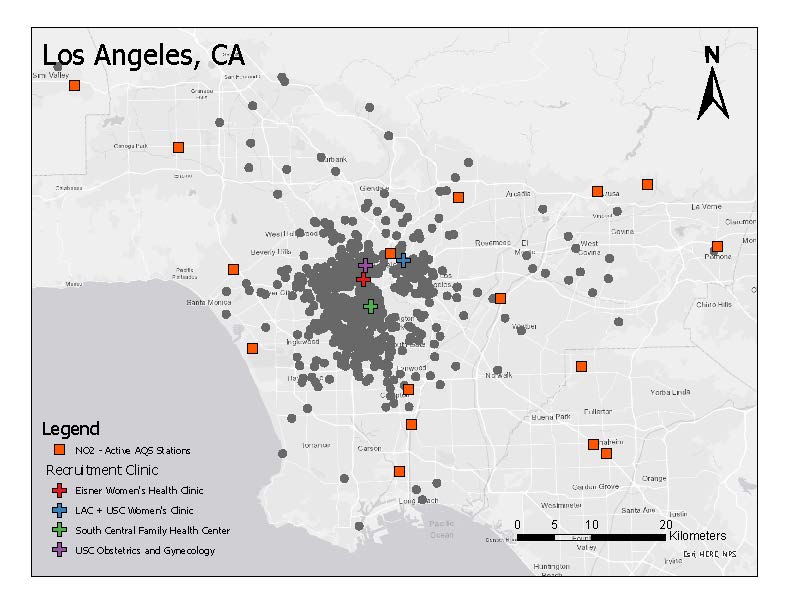

Supplement: Supplementary file 1 — Additional file 1: Supplement Figure 1. Monitoring network density for nitrogen dioxide (NO2) in Los Angeles, CA. [file 12940_2021_807_MOESM1_ESM.docx]
