# Supplementary material for: Prenatal ambient air pollution and maternal depression at 12 months postpartum in the MADRES pregnancy cohort
Source: Environ Health. 2021 Nov 27;20:121. doi: 10.1186/s12940-021-00807-x (PMC8626870; doi:10.1186/s12940-021-00807-x)

**Supplement Figure 2. Distribution of Scores on the Centers for Epidemologic Studies-Depression (CES-D) Scale at 12-Months Postpartum**


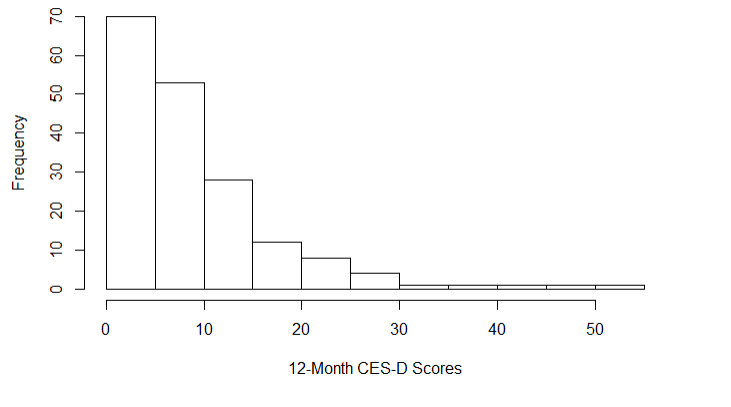

Supplement: Supplementary file 2 — Additional file 2: Supplement Figure 2. Distribution of Scores on the Center for Epidemologic Studies-Depression (CES-D) Scale at 12-Months Postpartum. [file 12940_2021_807_MOESM2_ESM.docx]
