# Supplementary material for: Prenatal ambient air pollution and maternal depression at 12 months postpartum in the MADRES pregnancy cohort
Source: Environ Health. 2021 Nov 27;20:121. doi: 10.1186/s12940-021-00807-x (PMC8626870; doi:10.1186/s12940-021-00807-x)
Supplement: Supplementary file 3 — Additional file 3: Supplement Figure 3. Spearman Correlations for each Ambient Pollutant by Trimester and Across Pregnancy. [file 12940_2021_807_MOESM3_ESM.docx]

| **Supplement Figure 3. Spearman Correlations for each Ambient Pollutant by Trimester and Across Pregnancy** | | | | | | | | |  |  |
| --- | --- | --- | --- | --- | --- | --- | --- | --- | --- | --- |
|  |  |  |  |  |  |  |  |  |  |  |
| **Nitrogen Dioxide (NO_2_), ppb** | | | | |  | **Ozone (O_3_), ppb** | | | | |
| **Averaging period** | **Trimester 1** | **Trimester 2** | **Trimester 3** | **Pregnancy Average** |  | **Averaging period** | **Trimester 1** | **Trimester 2** | **Trimester 3** | **Pregnancy Average** |
| **Trimester 1** | 1 |  |  |  |  | **Trimester 1** | 1 |  |  |  |
|  |  |  |  |  |  |  |  |  |  |  |
|  | N=179 |  |  |  |  |  | N=179 |  |  |  |
| **Trimester 2** | 0.16 | 1 |  |  |  | **Trimester 2** | 0.13 | 1 |  |  |
|  | p=0.03 |  |  |  |  |  | p=0.09 |  |  |  |
|  | N=179 | N=180 |  |  |  |  | N=179 | N=180 |  |  |
| **Trimester 3** | -0.64 | -0.06 | 1 |  |  | **Trimester 3** | -0.76 | -0.08 | 1 |  |
|  | p<0.0001 | p=0.44 |  |  |  |  | p<0.0001 | p=0.28 |  |  |
|  | N=178 | N=179 | N=179 |  |  |  | N=178 | N=179 | N=179 |  |
| **Pregnancy Average** | 0.43 | 0.74 | 0.14 | 1 |  | **Pregnancy Average** | 0.32 | 0.83 | 0.03 | 1 |
|  | p<0.0001 | p<0.0001 | p=0.06 |  |  |  | p<0.0001 | p<0.0001 | p=0.74 |  |
|  | N=179 | N=180 | N=179 | N=180 |  |  | N=179 | N=180 | N=179 | N=180 |
|  |  |  |  |  |  |  |  |  |  |  |
| **Particulate Matter <2.5 microns (PM_2.5_), µg/m³** | | | | |  | **Particulate Matter <10 microns, (PM_10_), µg/m³** | | | | |
| **Averaging period** | **Trimester 1** | **Trimester 2** | **Trimester 3** | **Pregnancy Average** |  | **Averaging period** | **Trimester 1** | **Trimester 2** | **Trimester 3** | **Pregnancy Average** |
| **Trimester 1** | 1 |  |  |  |  | **Trimester 1** | 1 |  |  |  |
|  |  |  |  |  |  |  |  |  |  |  |
|  | N=180 |  |  |  |  |  | N=179 |  |  |  |
| **Trimester 2** | 0.02 | 1 |  |  |  | **Trimester 2** | 0.27 | 1 |  |  |
|  | p=0.80 |  |  |  |  |  | p<0.01 |  |  |  |
|  | N=180 | N=180 |  |  |  |  | N=179 | N=180 |  |  |
| **Trimester 3** | -0.51 | 0.02 | 1 |  |  | **Trimester 3** | -0.27 | 0.31 | 1 |  |
|  | p<0.0001 | p=0.76 |  |  |  |  | p<0.01 | p<0.0001 |  |  |
|  | N=179 | N=179 | N=179 |  |  |  | N=178 | N=179 | N=179 |  |
| **Pregnancy Average** | 0.22 | 0.64 | 0.47 | 1 |  | **Pregnancy Average** | 0.44 | 0.81 | 0.61 | 1 |
|  | p<0.01 | p<0.0001 | p<0.0001 |  |  |  | p<0.0001 | p<0.0001 | p<0.0001 |  |
|  | N=180 | N=180 | N=179 | N=180 |  |  | N=179 | N=180 | N=179 | N=180 |
