# Supplementary material for: Prenatal ambient air pollution and maternal depression at 12 months postpartum in the MADRES pregnancy cohort
Source: Environ Health. 2021 Nov 27;20:121. doi: 10.1186/s12940-021-00807-x (PMC8626870; doi:10.1186/s12940-021-00807-x)
Supplement: Supplementary file 4 — Additional file 4: Supplement Table 1. Demographic characteristics in the full MADRES cohort and the current analytic sample. Supplement Table 2. Association of Prenatal Ambient Air Pollutants with Maternal Depression at 12 Months Postpartum, Additionally Adjusted for CalEnviroScreen Population Vulnerability Score. Supplement Table 3. Association of Prenatal Ambient Air Pollutants with Maternal Depression at 12 Months Postpartum, Additionally Adjusted for 12 Month Annual Average Postpartum Ambient Air Pollution. [file 12940_2021_807_MOESM4_ESM.docx]

| **Supplement Table 1. Demographic characteristics in the full MADRES cohort and the current analytic sample** | | | |
| --- | --- | --- | --- |
|  |  |  |  |
|  |  | **Full MADRES Cohort** | **Analytic Sample** |
|  |  | **N=842** | **N=180** |
|  |  | **Mean (SD)**  **or N (%)** | **Mean (SD)**  **or N (%)** |
|  |  |  |  |
| **Age at Enrollment (years)** |  | 28.4 (6.1) | 29.8 (6.0) |
|  |  |  |  |
| **Ethnicity and Nativity** |  |  |  |
|  | Non-Hispanic | 172 (20.4) | 36 (20.0) |
|  | US-Born Hispanic | 232 (27.6) | 64 (35.6) |
|  | Foreign Born (Mexico) | 169 (20.1) | 40 (22.2) |
|  | Foreign Born (All Other) | 101 (12.0) | 31 (17.2) |
|  | Missing / Refused to Answer | 168 (20.0) | 9 (5.0) |
| **Household Income** |  |  |  |
|  | Less than $30,000 | 356 (42.3) | 91 (50.5) |
|  | $30,000 to $49,999 | 82 (9.7) | 22 (12.2) |
|  | $50,000 or greater | 77 (9.2) | 28 (15.6) |
|  | Don't Know | 266 (31.6) | 38 (21.1) |
|  | Missing / Refused to Answer | 61 (7.2) | 1 (0.6) |
| **Educational Status** | |  |  |
|  | Completed 12th Grade or below | 442 (52.5) | 94 (52.5) |
|  | Some College or Tech School | 204 (24.2) | 43 (23.9) |
|  | College Graduate or Above | 118 (14.1) | 42(23.3) |
|  | Missing / Refused to Answer | 78 (9.3) | 1 (0.6) |
| **Marital Status** |  |  |  |
|  | Married | 202 (24.0) | 60 (33.3) |
|  | Living together | 252 (29.9) | 71 (39.4) |
|  | Never married, single | 146 (17.3) | 31 (17.2) |
|  | Divorced or separated | 20 (2.4) | 5 (2.8) |
|  | Missing / Refused to Answer | 222 (26.4) | 13 (7.2) |
| **Parity** |  |  |  |
|  | First-born | 237 (28.1) | 65 (36.1) |
|  | Second-born | 201 (23.9) | 48 (26.7) |
|  | Third-born or more | 205 (24.3) | 58 (32.2) |
|  | Missing/Refused to Answer | 199 (23.6) | 9 (5.0) |
| **Depression Diagnosis** | |  |  |
|  | Not Recorded on EMR | 783 (93.0) | 159 (88.3) |
|  | Yes-Recorded on EMR | 59 (7.0) | 21 (11.7) |
|  |  |  |  |

| **Supplement Table 2. Association of Prenatal Ambient Air Pollutants with Maternal Depression at 12 Months Postpartum, Additionally Adjusted for CalEnviroScreen Population Vulnerability Score** | | | | | | | |
| --- | --- | --- | --- | --- | --- | --- | --- |
| **Pollutant** | **Exposure**  **Averaging Period** | **Model 1: Unadjusted** | | **Model 2: Adjusted^b^** | | **Model 3: Adjusted^b^ Plus CalEnviroScreen Population Characteristics Score^c^** | |
|  |  | **N** | **OR^a^ (95% CI)** | **N** | **OR (95% CI)** | **N** | **OR (95% CI)** |
| **NO_2_ (ppb)** | |  |  |  |  |  |  |
|  | Trimester 1 | 179 | 1.02 (0.68 , 1.53) | 179 | 1.29 (0.70 , 2.40) | 179 | 1.20 (0.64, 2.26) |
|  | Trimester 2 | **180** | **1.82 (1.22 , 2.73)**** | **180** | **2.63 (1.41 , 4.89)**** | **180** | **2.63 (1.40, 4.93)**** |
|  | Trimester 3 | 179 | 1.03 (0.69 , 1.53) | 179 | 0.91 (0.55 , 1.49) | 179 | 0.91 (0.55, 1.49) |
|  | Across Pregnancy | **180** | **1.58 (1.05 , 2.37)*** | **180** | **2.04 (1.13 , 3.69)*** | **180** | **1.93 (1.06, 3.51)*** |
| **O_3_ (ppb)** |  |  |  |  |  |  |  |
|  | Trimester 1 | 179 | 0.89 (0.60 , 1.33) | 179 | 0.60 (0.31, 1.16) | 179 | 0.64 (0.33, 1.24) |
|  | Trimester 2 | 180 | 0.75 (0.51 , 1.12) | 180 | 0.68 (0.35, 1.32) | 180 | 0.70 (0.36, 1.37) |
|  | Trimester 3 | 179 | 1.14 (0.76, 1.70) | 179 | 1.48 (0.84, 2.63) | 179 | 1.46 (0.82, 2.60) |
|  | Across Pregnancy | 180 | 0.87 (0.57 , 1.32) | 180 | 0.88 (0.53, 1.47) | 180 | 0.91 (0.55, 1.52) |
| **PM_2.5_ (µg/m^3^)** | |  |  |  |  |  |  |
|  | Trimester 1 | 180 | 0.79 (0.52 , 1.20) | 180 | 0.80 (0.47, 1.35) | 180 | 0.79 (0.47, 1.32) |
|  | Trimester 2 | 180 | 1.40 (0.95 , 2.06) | **180** | **1.56 (1.01, 2.42)*** | 180 | 1.48 (0.96, 2.30) |
|  | Trimester 3 | 179 | 0.94 (0.63 , 1.41) | 179 | 0.93 (0.57, 1.51) | 179 | 0.86 (0.53, 1.40) |
|  | Across Pregnancy | 180 | 1.11 (0.75 , 1.64) | 180 | 1.33 (0.83 , 2.15) | 180 | 1.22 (0.75, 1.99) |
| **PM_10_ (µg/m^3^)** | |  |  |  |  |  |  |
|  | Trimester 1 | 179 | 0.88 (0.59 , 1.31) | 179 | 0.69 (0.37 , 1.28 ) | 179 | 0.65 (0.34, 1.25) |
|  | Trimester 2 | 180 | 1.34 (0.92 , 1.97) | 180 | 1.58 (0.97 , 2.56 ) | 180 | 1.59 (0.95, 2.66) |
|  | Trimester 3 | 179 | 1.03 (0.69 , 1.53) | 179 | 1.02 (0.58 , 1.80 ) | 179 | 0.96 (0.54, 1.73) |
|  | Across Pregnancy | 180 | 1.17 (0.79, 1.73) | 180 | 1.27 (0.80 , 2.04 ) | 180 | 1.24 (0.77, 2.02) |
| ^a^All odds ratios (OR) are scaled to one SD in exposure over the averaging period. | | | | | | | |
| ^b^Adjusted for recruitment site, maternal age, ethnicity by nativity, household income, education, air conditioning use during pregnancy, previous history of depression, and average temperature over the exposure averaging period. | | | | | | | |
| ^c^ Models additionally adjusted for CalEnviroscreen Population Characteristics Score assigned to study entry address. | | | | | | |  |
| *p<0.05; **p<0.01 | | | | | | | |

| **Supplement Table 3. Association of Prenatal Ambient Air Pollutants with Maternal Depression at 12 Months Postpartum, Additionally Adjusted for 12 Month Annual Average Postpartum Ambient Air Pollution** | | | | | | | |
| --- | --- | --- | --- | --- | --- | --- | --- |
| **Pollutant** | **Exposure Averaging Period** | **Model 1: Unadjusted** | | **Model 2: Adjusted^b^** | | **Model 3: Adjusted^b^ Plus Annual Average Postpartum Pollutant Levels^c^** | |
|  |  | **N** | **OR^a^ (95% CI)** | **N** | **OR (95% CI)** | **N** | **OR (95% CI)** |
| **NO_2_ (ppb)** |  |  |  |  |  |  |  |
|  | Trimester 1 | 179 | 1.02 (0.68 , 1.53) | 179 | 1.29 (0.70 , 2.40) | 179 | 1.29 (0.70, 2.39) |
|  | Trimester 2 | **180** | **1.82 (1.22 , 2.73)**** | **180** | **2.63 (1.41 , 4.89)**** | **180** | **2.70 (1.43, 5.12)**** |
|  | Trimester 3 | 179 | 1.03 (0.69 , 1.53) | 179 | 0.91 (0.55 , 1.49) | 179 | 0.91 (0.55, 1.50) |
|  | Across Pregnancy | **180** | **1.58 (1.05 , 2.37)*** | **180** | **2.04 (1.13 , 3.69)*** | **180** | **2.05 (1.13, 3.69)*** |
| **O_3_ (ppb)** |  |  |  |  |  |  |  |
|  | Trimester 1 | 179 | 0.89 (0.60 , 1.33) | 179 | 0.60 (0.31, 1.16) | 179 | 0.60 (0.31, 1.16) |
|  | Trimester 2 | 180 | 0.75 (0.51 , 1.12) | 180 | 0.68 (0.35, 1.32) | 180 | 0.69 (0.36, 1.33) |
|  | Trimester 3 | 179 | 1.14 (0.76, 1.70) | 179 | 1.48 (0.84, 2.63) | 179 | 1.51 (0.85, 2.69) |
|  | Across Pregnancy | 180 | 0.87 (0.57 , 1.32) | 180 | 0.88 (0.53, 1.47) | 180 | 0.89 (0.53, 1.48) |
| **PM_2.5_ (µg/m^3^)** |  |  |  |  |  |  |  |
|  | Trimester 1 | 180 | 0.79 (0.52 , 1.20) | 180 | 0.80 (0.47, 1.35) | 180 | 0.79 (0.45, 1.38) |
|  | Trimester 2 | 180 | 1.40 (0.95 , 2.06) | **180** | **1.56 (1.01, 2.42)*** | 180 | **1.58 (1.02, 2.45)*** |
|  | Trimester 3 | 179 | 0.94 (0.63 , 1.41) | 179 | 0.93 (0.57, 1.51) | 179 | 0.93 (0.57, 1.51) |
|  | Across Pregnancy | 180 | 1.11 (0.75 , 1.64) | 180 | 1.33 (0.83 , 2.15) | 180 | 1.41 (0.85, 2.33) |
| **PM_10_ (µg/m^3^)** |  |  |  |  |  |  |  |
|  | Trimester 1 | 179 | 0.88 (0.59 , 1.31) | 179 | 0.69 (0.37 , 1.28 ) | 179 | 0.69 (0.36, 1.32) |
|  | Trimester 2 | 180 | 1.34 (0.92 , 1.97) | 180 | 1.58 (0.97 , 2.56 ) | 180 | 1.56 (0.95, 2.56) |
|  | Trimester 3 | 179 | 1.03 (0.69 , 1.53) | 179 | 1.02 (0.58 , 1.80 ) | 179 | 0.97 (0.54, 1.77) |
|  | Across Pregnancy | 180 | 1.17 (0.79, 1.73) | 180 | 1.27 (0.80 , 2.04 ) | 180 | 1.24 (0.77, 2.00) |
| ^a^All odds ratios (OR) are scaled to one SD in exposure over the averaging period | | | | | | | |
| ^b^Adjusted for recruitment site, maternal age, ethnicity by nativity, household income, education, air conditioning use during pregnancy, previous history of depression, and average temperature over the exposure averaging period. | | | | | | | |
| ^c^ Each model was additionally adjusted for 12 month average ambient pollution level for each pollutant from birth to 12 months postpartum. | | | | | | | |
|  | | | | | | | |
